# Experimental Calibration of a Homogeneous Substitute Material Model for Reinforced High-Performance Concrete Modeling

**DOI:** 10.3390/ma16145056

**Published:** 2023-07-17

**Authors:** Jarosław Siwiński, Anna Szcześniak, Katarzyna Kubiak, Adam Stolarski

**Affiliations:** 1 Faculty of Civil Engineering and Geodesy, Military University of Technology, 2 Gen. Sylwestra Kaliskiego Street, 00-908 Warsaw, Poland; anna.szczesniak@wat.edu.pl (A.S.); adam.stolarski@wat.edu.pl (A.S.); 2 Remote Sensing Department, Center of Unmanned Technologies, Łukasiewicz-Institute of Aviation, 110/114 Krakowska Avenue, 02-256 Warsaw, Poland; katarzyna.kubiak@ilot.lukasiewicz.gov.pl

**Keywords:** homogeneous substitute material, numerical analysis, reinforced concrete modeling, homogenization, HSC, UHPC

## Abstract

The purpose of this work was to develop a substitute material model for the analysis of reinforced concrete structures. This paper presents proposals to solve the problem of limited calculation time, both to perform simulation models and to perform effective numerical or analytical analyses of structural elements in order to achieve results consistent with experimental results. Achieving this aim is conditional upon the determination of the material model parameters, taking into account the type of structure, the system of reinforcement, and the static strength–deformation parameters of the component materials. A universal procedure is proposed for determining the parameters of the substitute material model on the basis of the homogenization function, in which the homogenization coefficient is assumed as being equal to the effective reinforcement ratio of real reinforced concrete structural elements. In addition, the introduction of a new concrete constraint coefficient to this procedure, which corresponds to the proportionality coefficient of biaxial to uniaxial compressive strength, is proposed. On the basis of the conducted comparative analyses, the possibility of using the hypothetical substitute material model for the design of building elements and structures was confirmed. The average values of the obtained results for individual research series did not differ from the experimental results by more than 8.5%, for both the numerical and analytical models.

## 1. Introduction

The behavior of the numerical model of a structural system depends strictly on the material model used, which is an integral part of the procedure for modeling the structure of a building. Performing a spatial analysis of the behavior of a structural element or the entire structure of a building requires a complex numerical model. The difficulty of spatial modeling of reinforced concrete elements using high-strength concrete (HSC) and ultra-high-performance concrete (UHPC) lies in the separate modeling of structural materials (concrete and reinforcing steel) with their contact conditions. With a large number of reinforcing bars, each of them must be defined by its interaction with concrete, which increases the number of nodes used in the computational analysis and the analysis time. Due to the difficulties in modeling the properties of complex structural systems with heterogeneous reinforced concrete structures, an approach based on the homogenization of reinforced concrete is used. 

In one study [1], the results of an analysis of the use of a homogenization model to design a reinforced slab are presented. The work uses an approach with the division of the slab height into layers with different strength parameters. This approach uses partial homogenization since the same parameters have not been determined for the entire height of the section. In addition, modeling of interlayer connections was introduced, which extends the entire design stage of structural elements. However, no study on the procedure using a model of a homogeneous substitute material presents a universal procedure for determining the static and strength parameters for this material. 

In another study [2], an approach to analysis based on the determination of a key structural element was determined. Based on the homogenization approach, modified mechanical strength parameters for the indicated type of element were determined. Then, using an iterative approach, the strength parameters were selected in such a way as to obtain similar results of the stress and strain distribution. The approach presented in the paper does not allow the use of universal design without the need to perform a detailed analysis of the structural elements. 

Modeling the behavior of structural elements with increased mechanical and strength parameters [3,4,5,6] requires the modification of material models used in typical structures made of plain concrete. 

Furthermore, in [7], the authors presented a model of a homogeneous substitute material for modeling reinforced concrete. The methodology is based on the theory of homogenization of concretes with so-called normal strengths. Modeling by applying the homogenization coefficient was used to create numerical models using the Abaqus 6.23 software, which were then verified against the experimental results of the reinforced concrete beam and plate. Very good convergence of the load–displacement curve was achieved. On the other hand, the authors of [8] presented verification of the substitute material model subjected to dynamic loads, where high convergence of results was also achieved, and the use of the dynamicity coefficient was proposed.

In another study [9], solutions in the form of a closed moment–curvature response of rectangular cross-sections based on homogenized nonlinear material were presented. The equivalent flexural strength of a beam under the four-point bending test was shown to under predict the experimental values by as much as 30%. The research found that when using the uniaxial compression model for fiber-reinforced concrete (FRC), it is necessary to introduce coefficients modifying the flexural strength of concrete in order to correlate the results of analytical and numerical analyses with experimental results. The value of the correlation coefficient was determined depending on the composition of the concrete mix and the type of fibers in the range of 1.25–1.38.

In [10], the authors presented an evaluation of the elastic properties of fiber-reinforced concrete with homogenization theory and finite element simulation. It was found that the theoretical homogenization values and the finite element simulated values of the modulus of elasticity and Poisson’s ratio of the fiber-reinforced concrete were in good agreement with the experimental values that verified the accuracy of the theoretical homogenization model and the finite element model.

The authors of [11] presented a modified finite element method for nonlinear analysis of 2D beam structures based on the generalized nonlinear constitutive law described in [12]. The paper presented the generalized nonlinear constitutive law to update the stiffness of the plate element; this method is mainly used for modeling steel elements, but it can be used for reinforced-concrete elements. The main assumption of the method is an approach consisting of reducing the bending, tensile, and shear stiffnesses of the structures. Another assumption of this method is to limit the number of calculations generated in typical engineering programs. The method presented in [11,12] was used to iteratively change the linear parameters in order to achieve results similar to the load–strain curve in the elastic–plastic range.

The authors of [13] presented the use of the homogenization method in a numerical procedure to estimate the elastic modulus of concrete using the Abaqus 6.23 software in order to offer a low-computational cost. In turn, the authors of [14] presented a method of modeling reinforced concrete structural elements using the theory of homogenization in the scope of its ability to treat interactions between rebars and concrete affected by the Alkali–Silica Reaction (ASR).

This study proposes a methodology based on the expected compressive strength of HSC and UHPC concretes. The basic formula for designing the compressive strength of concrete is Feret’s law, which relates the compressive strength of high-strength concrete (HSC) (fluid consistency less than 0.4 *w*/*c*) to the water–cement ratio, the silica–cement ratio, cement class, and aggregate characteristics [15]. This relationship, among others, has been modified and used in [16,17]. The most commonly used model for determining the aggregate composition is the model of Andreasen and Andersen, which was modified by Funk and Dinger and described in [18,19,20].

The main purpose of this work is to determine a universal methodology for designing the parameters of the substitute material model, which can be used for modeling structural elements made of ordinary concretes as well as HSC and UHPC concretes. The proposed procedure makes it possible to determine the parameters of the substitute material using any concrete model, both in numerical and analytical versions. The presented homogenization function allows the determination of modified strength and deformation parameters for characteristic points on the stress–strain curve, and it can also be used for other types of concrete mixes and types of reinforcement, including nonmetallic ones. In the current literature, an approach based on the theory of homogenization used for modeling and designing structural elements and entire buildings on a macro scale has not been identified. The authors of this study did not find, in the existing literature, an approach based on the homogenization of reinforced concrete structures presented in a similar way to the proposed method. Typically, the available studies refer to the prediction of specific parameters, such as the modulus of elasticity and Poisson’s ratio. However, the determination of all characteristic points of the model on the stress–strain curve has not been presented. Therefore, in our work, the main emphasis was placed on comparison with the experimental results of other authors.

## 2. Method of Characterization of Homogeneous Substitute Material

### 2.1. Homogeneous Substitute Material Model

Based on the homogenization theory described for ordinary-strength concretes in works [7,8], a homogenous substitute material model for high-strength and ultra-high-strength concretes was determined, depending on the amount of fiber reinforcement used.

The concrete model implemented in the Abaqus software [21], developed by Lubliner et al. [22] and modified by Lee and Fenvs [23], was used in our research.

**Figure 1:**
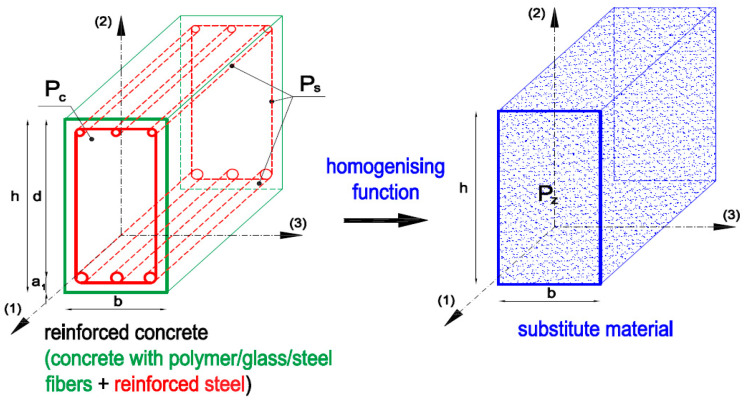
Scheme of the homogenization of reinforced concrete.

It should be emphasized that the presented homogenization methodology can be applied to all material models in order to modify strength and deformation parameters. The method of dividing the height of structural elements into layers allows for the elements to be modeled in a more accurate way; however, we wanted to propose a method that simplifies the entire modeling procedure as much as possible. The proposed analytical model presented in this study is independent and shows high convergence with experimental results. A method with the separation of layers with different parameters is presented in [24] on modeling shield elements. The principle of homogenization is shown in Figure 1 and Formula (1):

(1) $$P_{h}=P_{c}+F_{h}\cdot P_{s}$$

Here,

$P_{h}$—parameters of substitute material after homogenization;

$P_{c}$—concrete/fiber-reinforced concrete parameters;

$P_{s}$—steel parameters;

$F_{h}$—homogenization coefficient determined on the basis of Formula (2), which was adopted as the effective reinforcement ratio of the reinforced concrete element.

(2) $$F_{h}=\rho_{eff,h}$$

Here,

$\rho_{eff,h}$—the effective reinforcement ratio.

In Figure 1, the symbols (1)–(3) denote any directions perpendicular to each other. The effective reinforcement ratio was assumed as the resultant function of varied reinforcement ratios $\rho_{i}=\left\{\rho_{1},\rho_{2},\rho_{3}\right\}$ in distinguished reinforcement directions *i* = 1, 2, 3 and varied dimensionless coefficients of yield stresses in this reinforcement $\varphi_{yi}=f_{yi}/f_{y,max}$. Reinforcing steel parameters are differentiated for the lower and upper longitudinal reinforcement as well as for the transverse reinforcement and are taken as a weighted average for the area of the reinforcing steel used. The effective reinforcement ratio is expressed by Formula (3):

(3) $$\rho_{eff,h}=\sqrt{{\left(\rho_{1}\cdot \varphi_{y1}\right)}^{2}+{\left(\rho_{2}\cdot \varphi_{y2}\right)}^{2}+{\left(\rho_{3}\cdot \varphi_{y3}\right)}^{2}},$$

**Figure 2:**
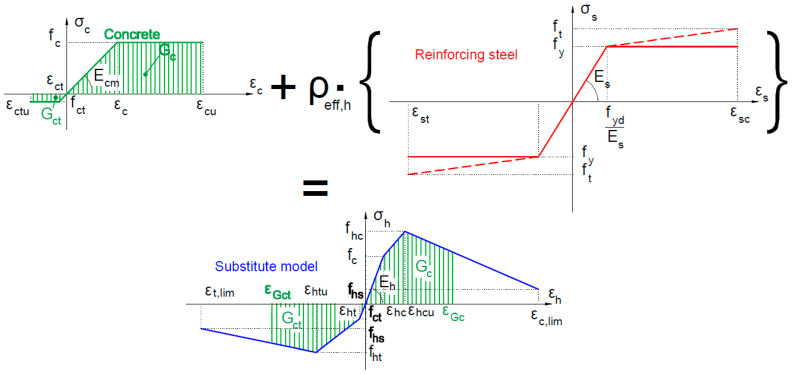
Schematic diagram of the substitute material model.

In addition, the homogenization function can be differentiated for the points of the stress–strain curve by introducing the yield strength and the ultimate tensile strength of the reinforcing steel at the point of the maximum compressive and tensile strength (*f_y_* or *f_t_*) to the substitute material model, depending on the adopted type of reinforcing steel (see Figure 2, red color).

In the substitute material model, it is also possible to take into account the strain rate effect by introducing the generalized dynamic strength coefficient *k_d_* > 1, which modifies the compressive and tensile strengths of a substitute material, for example, as shown in Formula (4) [8]:

(4) $$f_{hc}^{d}=k_{d}f_{hc},\;f_{ht}^{d}=k_{d}f_{ht},\;f_{hb}^{d}=k_{d}f_{hb},\;f_{hb}=\varphi_{ce}f_{hc}\;$$

where

$f_{hc}$—initial compressive strength; 

$f_{ht}$—initial tensile strength;

$f_{hb}$—biaxial compression for substitute model;

$\varphi_{ce}=1.16-1.2$—concrete constraint coefficient;

$k_{d}$ = 1.15—generalized dynamic strength coefficient. 

The schematic diagram of a substitute model is presented in Figure 2. The specific energy of compressive deformation *G_c_* and tensile deformation *G_ct_* are indicated in Figure 2 with a green color. These surfaces constitute the area under the stress–strain force diagram for concrete for the tension and compression zones, respectively. The strains *ε_Gc_* and *ε_Gct_* correspond to the surface area of the energy *G_c_* and *G_ct_*, and are taken as limit strains of concrete failure in compression and tension. In this way, we can determine the area of potential failure of finite elements in the model as the point of failure in the model. 

The following homogenized model parameters are specified:

*P_h_* = (*f_c_*—the initial concrete compressive strength; *f_ct_*—the initial concrete tensile strength; *f_hc_*—the maximum compressive strength; *f_ht_*—maximum tensile strength; *f_hs_*—the minimum compressive/tensile strength; *E_h_*—deformation modulus; ν*_s_*—coefficient of transversal deformability; and *γ_h_*—specific mass).

The following strain limits describe the homogenized model:

*ε_h_* = (*ε_hc_* = *f_c_*/*E_h_*—the initial elastic strain in compression; *ε_ht_* = *f_ct_*/*E_h_*—the initial elastic strain in tension; *ε_hcu_*—the strain corresponding to the maximum compressive strength; *ε_htu_*—the strain corresponding to the maximum compressive strength; *ε_c,lim_*—the limit strain corresponding to the minimum compressive strength (at buckling of rebar in reinforced concrete structure); and *ε_t,lim_*—the strain limit corresponding to the minimum tensile strength (at rupture).

The reference model of concrete was described in detail in [7,22,23].

**Figure 3:**
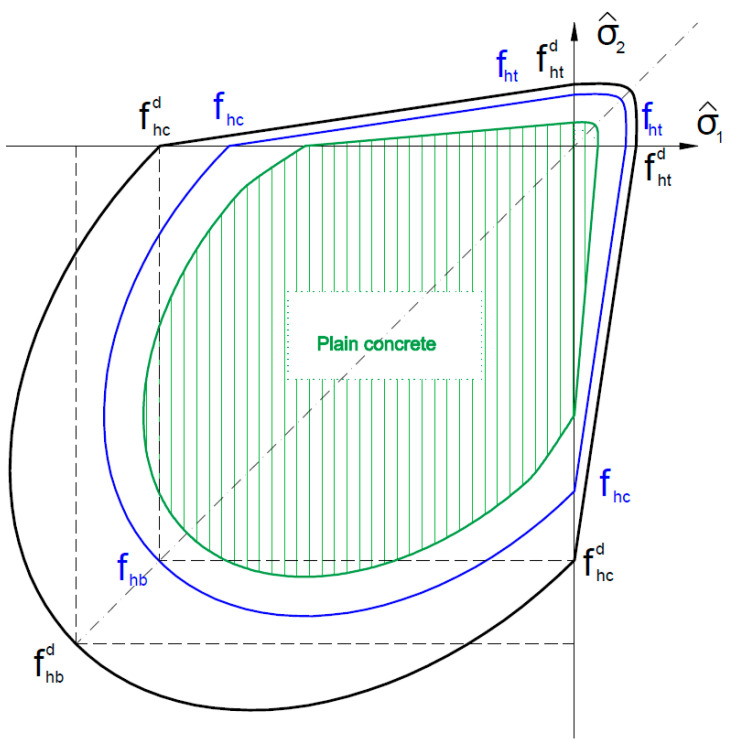
The limit plasticity function in the principal stresses plane.

In Figure 3, the limit plasticity function in the principal stresses plane (${\hat{\sigma }}_{1}$, ${\hat{\sigma }}_{2}$) is presented.

### 2.2. Substitute Material Modeling Procedure

In the developed procedure, the compressive strength of concrete was determined on the basis of Formula (5) [16,17] using the guidelines contained in [16]:

(5) $$f_{Lt}=\frac{k_{k}k_{fr}k_{sz}{k_{t}f_{cem}}_{}}{{\left[1+\frac{1.4\cdot \frac{W}{C+0.22m_{s}}}{1.4-0.4\cdot \exp\;(-11\cdot \frac{m_{s}}{C})}\right]}^{2}},$$

where

*f_Lt_*—the compressive strength of the concrete at the time of t days (MPa); *f_cem_*—the strength of the cement as measured on ISO mortar or design value (MPa); *k_k_* = 3.0 *+* (*ρ_B_* − *B_s_*)/*ρ_B_*—the aggregate coefficient, where *B_s_* is the number of binders and aggregates lower than 0.2 mm in size per kg in 1 m^3^ of concrete, and *ρ_B_* is the bulk density of all the aggregates in the sample (kg/m^3^); *k_fr_* = *exp*(0.034*ρ_S_*)—the reinforcing-fiber coefficient, where *ρ_S_* is the percentage ratio of steel-fiber mass to the mass of the cement (%), hereinafter *k_fr_* = 1.0 for *ρ_S_* = 0.0 is taken; *k_sz_*—the specimen shape and size coefficient (according to [13]); $k_{t}=\left[1-exp\left(-{\left(\frac{t-0.9}{3}\right)}^{0.6}\right)\right]$—the sample-curing-time coefficient where t is the sample-curing time (days); *W*/(*C +* 0.22*m_s_*)—the total amount of water included in all the mixture elements (including the water in the SP) relative to the amount of cement with 0.22 microsilica fume amount; and *m_s_*/*C*—the ratio of the microsilica fume to cement.

The procedure was carried out in such a way as to avoid the need to carry out experimental tests on standardized samples. The flexural tensile strength of concrete was determined on the basis of Formula (6) and Table 1. The data in Table 1 were determined on the basis of [16,25,26,27,28]:

(6) $$f_{ct,fl}=\rho_{cfl}\cdot f_{Lt},$$

where

$\rho_{cfl}$—the ratio of concrete compressive strength to concrete tensile strength. 

**Table 1:** The value of the coefficient *ρ_cfl_* depending on the amount of fiber reinforcement.

Percentage of Fiber Reinforcement [%]	${\rho c}_{fl}$
0	0.154
1	0.194
2	0.234
3	0.25
4	0.266

In the next step, the value of concrete tensile strength was determined according to Formula (7) in which the influence of the beam height was taken into account [29]:

(7) $$f_{ct}=\frac{0.4\cdot f_{ct,fl}}{1.6-\frac{h}{1000}},$$

where

h—beam height.

The elastic modulus *E_c_* is determined on the basis of Formula (8) [29]:

(8) $$E_{c}={22(0.1\cdot f_{Lt})}^{0.3}$$

The strain *ε_c_* at reaching the maximum compressive strength is determined on the basis of Formula (9):

(9) $$\varepsilon_{c}=\left[2.0+0.085{\left(f_{Lt}-50\right)}^{0.53}\right]\cdot k_{fr}$$

The ultimate strain *ε_cu_* for compressive strength is determined on the basis of Formula (10):

(10) $$\varepsilon_{cu}=\left[2.6+35{\left[0.01\left(90-f_{Lt}\right)\right]}^{4}\right]\cdot k_{fr}$$

The maximum (*ε_ct_*) and ultimate (*ε_ctu_*) tensile strains were determined according to Formulas (11) and (12), respectively.

(11) $$\varepsilon_{ct}=\frac{f_{ct}}{E_{c}},$$

(12) $$\varepsilon_{ctu}=10.0\;T_{c}\;\varepsilon_{ct},$$

where

$T_{c}$—ductility parameter from the closed interval [0.6, 1.0]; for fiber concrete, this is equal to 1.0 [30].

### 2.3. Analytical Substitute Material Model

**Figure 4:**
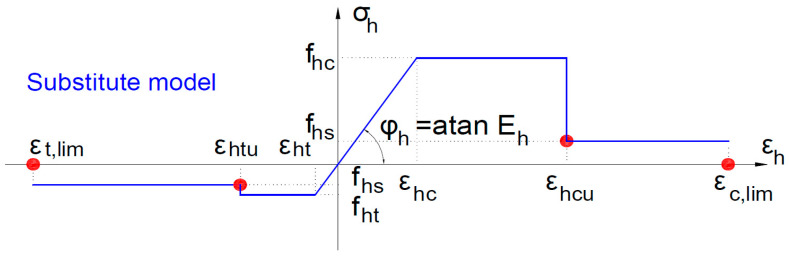
Schematic diagram of the substitute material model for analytical calculations.

As a substitute material model for analytical calculations, a deformation model was adopted in which all deformation and strength parameters were replaced with parameters determined in accordance with the assumptions of the homogenization method according to Formula (1). The schematic diagram of the substitute material model for analytical calculations is shown in Figure 4.

**Figure 5:**
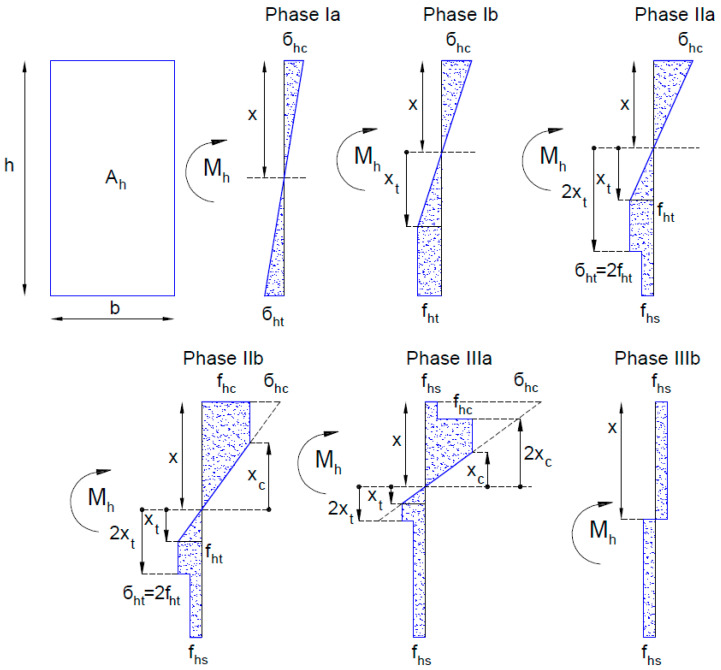
Diagrams of stresses in work phases of a cross-section made of the substitute material.

For the application and verification of the substitute material model in analytical calculations, the moment–curvature relationship is presented. The moment–curvature relationship for the cross-section made of substitute material was determined on the basis of the analysis of work phases of a uniform bending cross-section, assuming an elastic–ideally plastic stress distribution with different compressive and tensile strengths of the material, and taking into account the unlimited deformability of the material for tensile strength, as shown in Figure 5. 

In phase Ia, the resistance of the cross-section is determined by the elastic stresses in the compression zone and the tension zone of the cross-section. In phase Ib, the stresses in the compression zone remain elastic, and the stresses in the tension zone reach the tensile strength of the replacement material. In phase IIa, the stresses in the compressed zone remain elastic, and the strains in the tension zone exceed the value of the tensile strain limit of the replacement material, and then stresses appear in the lower cross-sectional zone equal to the minimum strength of the replacement material. In phase IIb, the compressive stresses reach the compressive strength of the replacement material. In phase IIIa, deformations in the compressed zone exceed the limit deformations of the substitute material for compression, and a zone with a minimum strength of the substitute material is formed. In phase IIIb, the theoretical limit load capacity of the cross-section is reached, which is determined by the rigid–plastic distribution of stresses equal to the minimum strength of the substitute material. In Table 2, static–strength relationships and formulas defining the bending moment and curvature in the working phases of a cross-section made of substitute material are shown.

## 3. Results and Discussion

### 3.1. Verification of the Numerical Substitute Model 

Verifications of the substitute material model in numerical calculations for ordinary concretes are presented in [7,8], while for HSC and UHPC concretes, calculations and comparative analysis with the experimental results are presented by Feng et al. [28].

Based on the test results presented in this paper, the parameters of the substitute material were determined in two variants. In variant 1, all parameters of the substitute material were determined on the basis of static and strength parameters being the results of the authors’ research. In variant 2, the parameters of the substitute material were determined based only on information on the materials, i.e., the cement used, the concrete recipe, and the steel grade. Other parameters were determined on the basis of dependencies indicated in this study, results for series B20-B30 are shown in Table 3. The designation B20 is consistent with the paper by Feng et al. [28] and means that it is UHPC concrete with 2.0% of reinforcing fibers. B20-S means that all strength and deformation parameters were determined on the basis of the authors’ procedure. Similarly, the symbols of the B30 and B30-S series are used, with the concrete compressive strength entered as $f_{c}$ and designed according to the authors’ own procedure as $f_{Lt}$.

We noticed that the compressive strength of concrete, according to the authors of experimental research [28] and our own results, differ by 0.5% for the B20 series and 1.5% for the B30 series, respectively. In turn, the tensile strength of concrete differs by 13.7% and 15.5% for the B20 and B30 series, respectively. The modulus differences are 3.8% for the B20 series and 0.2% for the B30 series. The values of strains at maximum stress, $f_{c}$ and $f_{ct}$, and limit strains for the B20 series differ by 22.4% and 18.6%, and for the B30 series, they differ by 10.3 and 13.3%. It should be noted, however, that the deformation values in the experimental tests were made for hook-shaped fibers, while the authors’ procedure is an average value for various types of fibers used in HSC and UHPC concretes. Despite major differences, it was found that this approximation is satisfactory and will have an impact on the course of the stress–strain curve and, to a lesser extent, on the point determining the strength of the beam.

**Figure 6:**
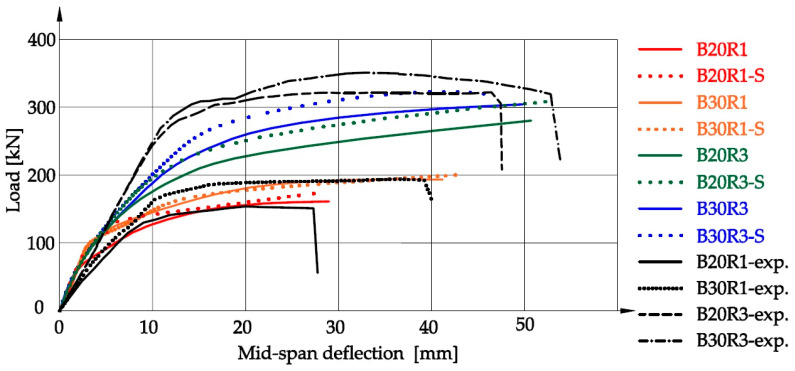
Numerical relationship load vs. mid-span deflection diagram.

Based on the determined parameters of the substitute material model, a numerical analysis was carried out using the Abaqus 6.23 software. Figure 6 shows the load–displacement diagrams for the B20R1, B20R3, B30R1, and B30R3 beams in variants 1 and 2, according to the results of experimental tests and the results of the authors’ own procedure. The results of the maximum load and maximum displacement at the center of the beam are shown in Table 4. It was found that the maximum difference in loading between the model made on the basis of the authors’ procedure and the results of experimental studies by Feng et al. [28] is 13.5% for the B20R1-S series. Differences for other series do not exceed 4.5%. The average value of the limit load results for the BXRY series (X = 20 or 30, Y = 1 or 3) in relation to the experimental results was 7.1%, while the corresponding average value for the BXRY-S series, developed on the basis of the procedure, was 5.55%. The average displacement results in the middle of the beam for the BXRY series differ from the experimental results of the authors [28] by 5.4%, while for the BXRY-S series, the results differ by 8.5%. The difference in numerical results according to the modeling of the substitute material on experimental data and determined according to our own procedures is 3.1% and may be caused by the type of fibers used in the concrete. At the current research stage, the procedure is a universal solution without distinguishing the shape of the fibers, which can have a significant impact on the results of deformations and, thus, on the course of the load–displacement diagram.

### 3.2. Verification of the Analytical Substitute Model

**Figure 7:**
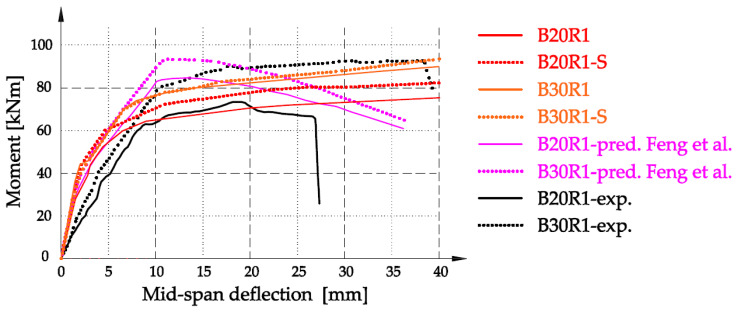
Analytical relationship moment vs. mid-span deflection diagram for series B20R1 and B30R1, in relation to the results [28].

Verifications of the numerical substitute model were made on the basis of a comparative analysis of the authors’ own results and the experimental results of Feng et al. [28]. In Figure 7, the results of the moment–mid-span deflection for series B20R1 and B30R1 are shown. We noticed that the results of analytical calculations based on the described analytical model show a high degree of convergence with the experimental results. The course of the B20R1 series curve is the closest result to the course of the experimental line. At the displacement point of 27 mm, the difference in the bending moment is only 6.4 kNm. It should be emphasized that the analytical model did not include the values of limit deformations representing the destruction of pure concrete, hence the course of the curve is extended in relation to the experimental result. A very good convergence of the moment result was obtained for the B20R3 series at the displacement point of 37.3 mm, assumed as the beam failure in the experiment. The B20R3-S series obtained exactly the same value, while for the B20R3 series the difference was 3.26 kNm. In relation to the results of the analytical model of the authors of [28], based on the model contained in the standard [31], the curves obtained were more similar to the experimental results.

**Figure 8:**
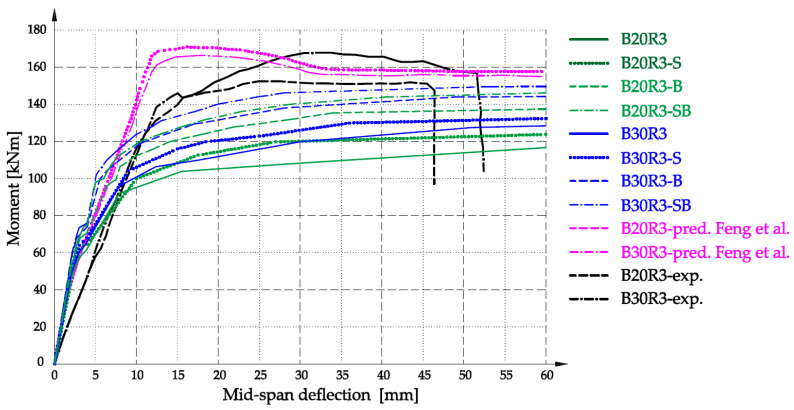
Analytical relationship moment vs. mid-span deflection diagram for series B20R3 and B30R3, in relation to the results [28].

In turn, Figure 8 shows the results of the moment–mid-span deflection curve for the B20R3 and B30R3 series. In the drawing, additional series marked as B20R3-B, B20R3-SB, B30R3-B, and B30R3-SB have been introduced, which were made with the substitute material achieving biaxial compression values, analogous to the numerical model, i.e., the values of compressive strength, tensile strength, and modulus of elasticity multiplied by 1.16, the concrete constraint coefficient. This procedure was adopted due to the large amount of longitudinal reinforcing steel, which stiffens the beam structure, forcing the concrete to work in a greater range of biaxial bending. In order to reflect this phenomenon, the changed strength parameters were introduced in the substitute material model by applying the concrete constraint coefficient $\varphi_{ce}$. 

The difference in the displacement point of 47.0 mm was 2.85 kNm for the B20R3-SB series, which was about 1.9% of the breaking force, while for the B20R3-B series, it was 11.1 kNm and 7.5%, respectively. The difference in the displacement point of 52.0 mm was 7.47 kNm for the B30R3-SB series, which was about 4.7% of the breaking force, while for the B30R3-B series it was 12.85 kNm and 8.2%, respectively. It should be emphasized, however, that according to the EC2 standard, the maximum permissible degree of reinforcement of structural elements is 4%. The R3 series analyzed in the article were marked as having 2.9% of reinforcement, while taking into account the upper reinforcement of 3.5%, which is close to the limit value. In most traditionally designed structural members, the economic reinforcement ratio is between 0.8% and 2.0%. In this respect, the convergence of the results according to the described procedure is the highest.

## 4. Conclusions

Based on the tests and analyses carried out, it was found that the procedure for designing a substitute material model allows for achieving satisfactory results in the modeling of structural elements. Results similar to experimental results, and similar approximation to analyses using other analytical or numerical models, confirm the possibility of using homogenization for structural elements made of HSC and UHPC concretes. The following conclusions emerge from the conducted analyses:

(1) Modeling the substitute material model according to the proposed procedure allows for the achievement of numerical results convergent with experimental results for reinforced elements made of UHPC concrete. The average results of displacements in the middle of the beam for the series using the experimental results of the concrete itself differ from the authors’ experimental results by 5.4%, while for the series in which all strength and deformation parameters were determined the results differ by 8.5%.
(2) The difference between the results using the analytical substitute model with the experimental results is in the range of 1.9% to 8.2%.
(3) In the case of structural elements with a large amount of longitudinal reinforcement, it is recommended to take into account the work of the substitute material in terms of biaxial compression by applying an additional multiplier called the concrete constraint coefficient with a value in accordance with the values in the range (1.16–1.2) adopted in the literature.
(4) The highest accuracy of displacement results equal to 1.9% was achieved for the B20R1 series using the results of experimental studies.
(5) The highest accuracy of the breaking load results equal to 0.5% was achieved for the B30R1 series using the results of experimental tests.
(6) The average displacement results for the R1 and R1-S series reached an accuracy of 5.1%, while for the R3 and R3-S series, it was 11.0%.

The presented results of this study confirm the high efficiency of the proposed method for the homogenization of reinforced concrete structures.

The modeling methodology based on the homogenization theory can also be applied to structural elements reinforced with the use of nonmetallic reinforcement because it consists of modifying the basic deformation and strength parameters of the concrete model used. The method can also be used to modify other material models for hybrid and composite elements.

The model can be used for any configuration of longitudinal and transversal reinforcement, but we must remember that structural elements and the entire building must be designed in accordance with applicable standards, which specify, among others, the minimum amount and shape of individual types of reinforcement.

## Figures and Tables

**Table 2:** Static–strength relationships and formulas defining the bending moment and curvature in the working phases of a cross-section made of substitute material.

**Phase**	**Ia**	**Ib**	**IIa**
Boundary condition	$0\leq \sigma_{ht}\leq f_{ht}$	$f_{ht}\leq \sigma_{ht}\leq {2f}_{ht}$	${\sigma_{hc,max,Ib}\leq \sigma }_{hc}\leq f_{hc}$
x	$x=\frac{h}{2}$	$x=h\frac{A\pm \sqrt{A}}{A-1}$, $0\leq x\leq x_{Ia,max}$ $A=2\frac{f_{ht}}{\sigma_{ht}}-\frac{{f_{ht}}^{2}}{{\sigma_{ht}}^{2}}$	$x=h\frac{f_{hs}}{\frac{1}{2}\sigma_{hc}-\frac{3}{2}\frac{{f_{ht}}^{2}}{\sigma_{hc}}+f_{hs}\left(1+2\frac{f_{ht}}{\sigma_{hc}}\right)}$
$x_{t}$	-	$x_{t}=(h-x)\frac{f_{ht}}{\sigma_{ht}}$	$x_{t}=x_{c}\frac{f_{ht}}{\sigma_{hc}}$
$\sigma_{hc}$	$I_{Ia}=\frac{bx^{3}}{3}+\frac{b(h-{x)}^{3}}{3}=\frac{bh^{3}}{12}$	$\sigma_{hc}=\sigma_{ht}\frac{x}{h-x}$	-
M	$M_{Ia}=\sigma_{ht}\frac{I_{Ia}}{h-x}$	$M_{Ib}=\frac{1}{3}\sigma_{hc}bx^{2}+\frac{1}{3}f_{ht}bx_{t}^{2}+\frac{1}{2}f_{ht}b(h-x-x_{t})(h-x+x_{t})$	$M_{IIa}=\frac{1}{3}\sigma_{hc}bx^{2}+\frac{11}{6}f_{ht}bx_{t}^{2}+\frac{1}{2}f_{hs}b(h-x-2x_{t})(h-x+{2x}_{t})$
κ	$\kappa_{Ia}=\frac{M_{Ia}}{E_{h}I_{Ia}}=\frac{\sigma_{ht}}{E_{h}(h-x)}$	$\kappa_{Ib}=\frac{\sigma_{ht}}{E_{h}(h-x)}$	$\kappa_{IIa}=\frac{\sigma_{hc}}{E_{h}x}$
**Phase**	**IIb**	**IIIa**	**IIIb**
Boundary condition	${f_{hc}\leq \sigma }_{hc}\leq {2f}_{hc}$	${0<x}_{c}\leq 0.5x_{IIb,max}$	${x_{IIIa,\;max}\geq x}_{c}\to 0$
x	$x=h\frac{f_{hs}}{f_{hc}-\frac{1}{2}\frac{{f_{hc}}^{2}}{\sigma_{hc}}-\frac{3}{2}\frac{{f_{ht}}^{2}}{\sigma_{hc}}+f_{hs}\left(1+2\frac{f_{ht}}{\sigma_{hc}}\right)}$	$x=\frac{h}{2}-(\frac{3}{4}\frac{f_{hc}}{f_{hs}}-1)x_{c}+(\frac{3}{4}\frac{f_{ht}}{f_{hs}}-1)x_{t}$	x=0.5h
$x_{t}$	$x_{t}=x\frac{f_{ht}}{\sigma_{c}}$	$x_{t}=x_{c}\frac{f_{ht}}{f_{hc}}$	-
$\sigma_{hc}$	-	$\sigma_{hc}=f_{hc}\frac{x}{x_{c}}>2f_{hc}$	-
M	$M_{IIb}=\frac{1}{3}f_{hc}bx_{c}^{2}+\frac{1}{2}f_{hc}b\left(x-x_{c}\right)\left(x+x_{c}\right)+\frac{11}{6}f_{ht}bx_{t}^{2}+\frac{1}{2}f_{hs}b(h-x-2x_{t})(h-x+{2x}_{t})$	$M_{IIIa}=\frac{11}{6}f_{hc}bx_{c}^{2}+\frac{1}{2}f_{hs}b\left(x-{2x}_{c}\right)\left(x+{2x}_{c}\right)+\frac{11}{6}f_{ht}bx_{t}^{2}+\frac{1}{2}f_{hs}b(h-x-2x_{t})(h-x-2x_{t})$	$M_{IIIb}=\frac{1}{2}f_{hs}bx^{2}+\frac{1}{2}f_{hs}b{\left(h-x\right)}^{2}=\frac{1}{4}f_{hs}bh^{2}$
κ	$\kappa_{IIb}=\frac{\sigma_{hc}}{E_{h}x}$	$\kappa_{IIIa}=\frac{\sigma_{hc}}{E_{h}x}=\frac{f_{hc}}{E_{h}x_{c}}$	${\kappa_{IIIa}\leq \kappa }_{IIIb}=\underset{x_{c}\to 0}{\lim}\kappa_{IIIa}\to \infty$

**Table 3:** The value of the coefficient $\rho_{cfl}$ depending on the amount of fiber reinforcement.

Concrete Marking	$f_{c}$ or $f_{Lt}$ [MPa]	$f_{ct}$ [MPa]	$E_{c}$ [GPa]	$\varepsilon_{c}$ [µε]	$\varepsilon_{cu}$ [µε]	$\varepsilon_{ct}$ [µε]	$\varepsilon_{ctu}$ [µε]
B20	125.4	7.9	45.1	2780	4088	156	2705
B20-S	124.8	9.35	46.9	3037	3340	210	2280
B30	128.4	10.5	47.4	2709	4313	189	3048
B30-S	130.4	10.43	47.5	3177	3910	240	2690

**Table 4:** Results of maximum load and maximum mid-span deflection of the beam.

Series Marking	Maximum Load [kN]	(BXRY or BXRY-S) /BXRY-exp. Load Difference [%]	Maximum Mid-Span Deflection [mm]	(BXRY or BXRY-S) /BXRY-exp. Deflection Difference [%]
B20R1	150	6.4	27.5	1.9
B20R1-S	160	13.5	28.5	5.6
B20R1-exp.	141	-	27.0	-
B20R3	280	−12.5	50.0	8.7
B20R3-S	315	1.6	52.0	21.7
B20R3-exp.	320	-	46.0	-
B30R1	194	−0.5	41.0	5.1
B30R1-S	200	2.6	42.0	7.7
B30R1-exp.	195	-	39.0	-
B30R3	305	−9.0	49.0	−5.8
B30R3-S	320	−4.5	48.0	−7.7
B30R3-exp.	335	-	52.0	-

## Data Availability

Not applicable.
